# Videofluoroscopy of the aerodigestive tract in *Phoca vitulina*: reshaping perspectives on translational medicine

**DOI:** 10.3389/fvets.2024.1412173

**Published:** 2024-07-18

**Authors:** Stacey A. Skoretz, Arlo Adams, A. Wayne Vogl, Stephen Raverty, Martin Haulena, Hillary Stahl, Camilla Dawson

**Affiliations:** ^1^School of Audiology and Speech Sciences, University of British Columbia, Vancouver, BC, Canada; ^2^Department of Critical Care Medicine, University of Alberta, Edmonton, AB, Canada; ^3^Department of Cellular and Physiological Sciences, Life Sciences Institute, University of British Columbia, Vancouver, BC, Canada; ^4^Animal Health Center, Ministry of Agriculture and Food, Abbotsford, BC, Canada; ^5^Vancouver Aquarium, Vancouver, BC, Canada; ^6^Vancouver Coastal Health, Vancouver, BC, Canada; ^7^University Hospitals Birmingham NHS Foundation Trust, Birmingham, United Kingdom; ^8^Institute of Clinical Sciences, College of Medical and Dental Sciences, University of Birmingham, Birmingham, United Kingdom

**Keywords:** *Phoca vitulina*, videofluoroscopy, swallowing, rehabilitation, upper aerodigestive tract, airway protection

## Abstract

Thousands of rescued harbor seals (*Phoca vitulina*) require rehabilitation worldwide. Many require resource intensive gavage feeding due to abandonment soon after birth. Little is known about seal swallowing, therefore, our primary objective was to determine the feasibility of conducting videofluoroscopic swallowing studies (VFS) on seal pups prior to their release. Secondarily, we propose swallowing phase descriptions. We adapted a VFS approach used in humans and our feasibility parameters included: bolus detection and consumption, and number of analyzable swallowing events. Unrestrained seals were imaged in a dry environment using a Siemens mobile c-arm fluoroscopy unit. Oral boluses were thawed herring injected with liquid barium suspension (105% w/v). Two independent raters described swallows using a standardized approach with results summarized descriptively. We successfully completed freely-behaving VFS with two infant seals (1 male: 8 wks, 3 d; 1 female: 5 wks, 3 d). Both consumed five boluses with six fully analyzable swallowing events. We describe four swallow phases: preparatory, prehension, oropharyngeal and esophageal. Airway protection likely occurs in two ways: (1) during the preparatory phase through modified corniculate cartilage contact with the glottis and (2) with soft palate contact to the base of tongue prior to swallow initiation. We have conducted a unique VFS approach on rehabilitated seals, prior to their release. We have described airway protection and suggest that swallowing is initiated earlier in the feeding process than described previously. This protocol success will afford: (1) collection of normative swallowing data, and (2) future knowledge translation from humans to seals.

## 1 Introduction

Over the past decade, thousands of infant harbor seals have been admitted to the Vancouver Aquarium’s Marine Mammal Rescue Centre (MMR) and other rehabilitation facilities worldwide (1). From 2012 to 2020, the MMR successfully rehabilitated and released 1,017 individuals – 80% of their harbor seal admissions (1). In a retrospective review of necropsies following natural death after stranding, the highest cause of mortality in seal pups was a lack of nutrition, with sub-adults/adults dying primarily from infections secondary to bacterial pneumonia (2). With the ultimate goal to return to the wild (3), stranded seals receive life sustaining medical care, management, and rehabilitation. Successful rehabilitation efforts are a vital component of population recovery across many locations (4), contributing to endangered seal species recovery (5, 6), as in the Mediterranean and Hawaiian monk seal, and environmental stabilization (4). As many rescued seal pups and neonates cannot feed on their own, they require frequent gavage (tube feeding) to ensure adequate nutrition and efficient medication delivery (7). To safeguard seal and rehabilitation team welfare, gavage requires animal restraint during tube placement and throughout the feeding process. Once permanent dentition is fully functional at the age of 4–6 weeks (8, 9), reliance on tube feeding is decreased through gradual weaning and introduction of a whole fish (oral) diet.

Weight gain is a key predictor of survival both after weaning and on release, thus transition to oral intake (weaning) must be efficient and effective (7, 10). To maintain seal health, the progression to independent oral intake occurs over several weeks in a multi-step, gradual process. At the MMR, rehabilitation staff engage in a systematic step-wise program where harbor seals are hand-fed herring with varying degrees of oral and tactile stimulation used to support its retention within the oral cavity (7). Based on the progression of the seal, the weaning/feeding approach is adjusted according to the animal need. For example, early stages may involve inserting a small herring directly into the oral cavity while holding the muzzle closed until a swallow is triggered a few times a day, while optimizing nutrition by tube. Later stages may involve simulating water movement in the tank to draw attention to herring presence, mirroring naturally occurring circumstances in which prey would appear in the wild. Once the seals are able to feed independently, they are transitioned to communal tanks feeding with others prior to release. While this conventional rehabilitation approach has proved effective for many centers over the years (7, 11–13), it is invasive and resource intensive. Adaptations to the rehabilitation process (14) may be informed by thorough investigation of upper aerodigestive tract (UAT) anatomy and physiology, specifically understanding clinically relevant events during the oral, pharyngeal and esophageal swallowing phases.

The respiratory tract (larynx, trachea, bronchi, and lungs) evolved as an outgrowth of the foregut, and develops embryologically as a diverticulum from the endodermal gut tube. The larynx is the cranial most part of the lower airway and exists at the interface between the pharynx and trachea. It is not surprising that the major function of the larynx is to act as a valve, or sphincter, that prevents food and liquid from entering the airway. Closure of the laryngeal inlet and apposition of mucosal folds within the laryngeal cavity effectively block the entrance to the airway during swallowing. In mammals generally, the vocal folds on the lateral walls of the laryngeal inlet have been further modified to produce sounds as air courses between them. These sounds can be modulated by changes in the position of the larynx in the neck (15–17), by skeletal muscle activity that changes the length and tension of the folds, by the addition of accessory tissues that change the mass of the folds (18), and by structures in and around the oral cavity which modify resonance. In general, the larynx demarcates the transition from an oral (19) to a respiratory tract (20). In harbor seals, the larynx is positioned high in the neck (21) relative to humans, however the superior edge of the structure is not anchored into the nasopharynx by a skeletal muscle sphincter like it is in odontocete cetaceans (22). In our earlier work (21), we described potential laryngeal modifications which may play a role in swallowing in the harbor seal – “the arytenoid and corniculate cartilages together form prong-like projections directed forward to form the posterior aspects of the lateral margins of the laryngeal inlet (p. 3).” As a result, further analyses of our *in vivo* imaging was conducted to better understand how the larynx and other UAT structures function during swallowing in harbor seals.

Previous work in pinnipeds has focused on feeding methods (23–25), ways in which the prey is moved from the environment to the oral cavity (e.g., filter, biting, and suction). Often, swallowing physiology in the harbor seal has been inferred through external observation of anatomical movements (26). In order to develop innovative feeding and swallowing rehabilitation, regardless of species, understanding UAT swallowing physiology according to clinically relevant phases is imperative. For some mammals, swallowing research has focused on acquired dysphagia due to pathology (27, 28), animal models for human applications (29) and/or establishing species specific imaging protocols (30). In the area of feeding and swallowing rehabilitation, studies including harbor seals have explored environmental enrichment (31) with limited work on adaptive feeding devices (14). To the best of our knowledge, interventions focusing on bridging therapies when transitioning to oral intake from tube feeding, have yet to be systematically explored in this species. In human pediatric rehabilitative medicine, well-developed programs exist where bridging therapies are systematically used during tube feeding, and as the infant transitions to oral intake (32) – these approaches have yet to be investigated in pinnipeds but require an understanding of their swallow early in development. To the best of our knowledge, *in vivo* radiological, dynamic imaging of oral, pharyngeal and esophageal function has not yet been investigated in seals. As a result, how to optimize tube feeding and/or weaning processes are empirical and whether approaches used in human medicine could be leveraged for use in seals remains unknown. To explore this hypothesis and to further report on our innovative upper aerodigestive tract anatomical (21) and physiological (33) line of inquiry in harbor seals, our primary objective was to determine the feasibility of conducting videofluoroscopic swallowing studies on seal pups. Our secondary objective was to develop swallowing phase descriptions in the harbor seal through observation of the oral, pharyngeal and esophageal swallowing phases during videofluoroscopy.

## 2 Materials and methods

### 2.1 Animals

We conducted this study at the University of British Columbia (UBC) Centre for Comparative Medicine (CCM, Vancouver, BC, Canada) in collaboration with the Vancouver Aquarium’s Marine Mammal Rescue Centre (MMR). Prior to release to the wild, we imaged a convenience sample of rehabilitated harbor seal pups (*Phoca vitulina*) using videofluroscopy. These seals were able to self-feed and had no evidence of previous clinical history of feeding and/or swallowing difficulties. In addition to the principal investigator (SAS), other attending personnel included those for animal health and welfare: a veterinarian (MH), two veterinary technicians, and an imaging technician. This study was approved by the UBC Animal Care Committee (A18-0252) and personnel conducting the protocol completed animal welfare training and ethical research approaches as per UBC requirement.

### 2.2 Videofluoroscopy

A videofluroscopic swallowing study (VFS) is a dynamic imaging technique used in standard human clinical practice to assess swallowing (from oral cavity to the esophagus) and diagnose dysphagia (34). We adapted a VFS approach used in humans for use in seals. A Siemens mobile c-arm fluoroscopy unit and plastic translucent tub were utilized for imaging. Upon arrival at the CCM, the seals were kept in kennels typically used for their transport and in a quiet, dark space. Seals were lifted into the imaging tubs by their attending veterinarian team. Imaging was conducted in the lateral plane and the seals were unrestrained and able to behave freely. They were not anaesthetized or sedated. Once the seal was placed in the tub and moving freely, a 10-min period was afforded prior to imaging so that the seal could acclimatize and investigate their surroundings (Supplementary Figure A: Figure S1). Veterinarian staff monitored the seals for any signs of distress or discomfort throughout the imaging. All personnel conducting imaging were wearing protective lead equipment not limited to lead aprons, thyroid shields, gloves (MH), and eyewear.

The food boluses consisted of whole (previously flash frozen) herring, prepared with a barium suspension. Barium is a radiopaque contrast agent conventionally employed during videofluoroscopy in human (34, 35) and animal medicine (27, 30). After thawing but before presentation to the seal, we injected the herring gills and swim bladders with liquid barium suspension (Liquid Polibar Plus® Barium Sulfate Suspension, 105% w/v, E-Z-EM Canada Inc.). Excess barium on the surface of the fish was removed by disposable towels. The attending veterinarian (MH), presented the food bolus to the seal by hand at muzzle level. Throughout each videofluoroscopy, five boluses were delivered. The swallow was imaged at 30 frames per second with lossless digital capture. The view for each analyzable swallow event included the anterior lips to the proximal esophagus with panning to the lower esophageal sphincter as able.

### 2.3 Analysis

Two raters (HS, JV) blinded to each other described the structural movements and major deglutition events across four proposed phases adapted from human imaging: preparatory, oral, pharyngeal and esophageal. They evaluated swallow physiology frame-by-frame using TIMS™ DICOM review software (TIMS Medical and Foresight Imaging LLC). Following their rating, the two raters were unblinded and disagreements regarding interpretation were resolved by consensus. Confirmation of all findings was conducted by a third expert rater (SAS). Collectively, these raters have extensive experience with videofluoroscopic evaluation of swallowing including standardized rating training (35). We summarized the results descriptively.

## 3 Results

We successfully conducted freely-behaving videofluoroscopic swallowing studies on two infant seals (1 male: 8 wks, 3 d; 1 female: 5 wks, 3 d). Prior to videofluoroscopy, the male was in rehabilitation for 8 weeks and the female for 2 weeks and 3 days. For each seal, five boluses were administered (67–90 g each), with 10 (N) recorded swallowing events. Total fluoroscopy exposure time for each individual study was approximately 3 min which included lower gastrointestinal imaging (not reported here). Because of seal movement, six swallowing events were fully analyzable (three for each seal), with four partially analyzable. To meet our criteria for full analyses, the radiographic image had to contain a full view of the head (including lips) and proximal esophagus during the bolus presentation and swallow. Following our analyses, we delineated the following distinct but overlapping swallowing phases: preparatory, prehension, oropharyngeal and esophageal. Our phase titles were modified from that which we originally proposed based on the observed swallowing events.

### 3.1 Preparatory phase

**Figure 1:**
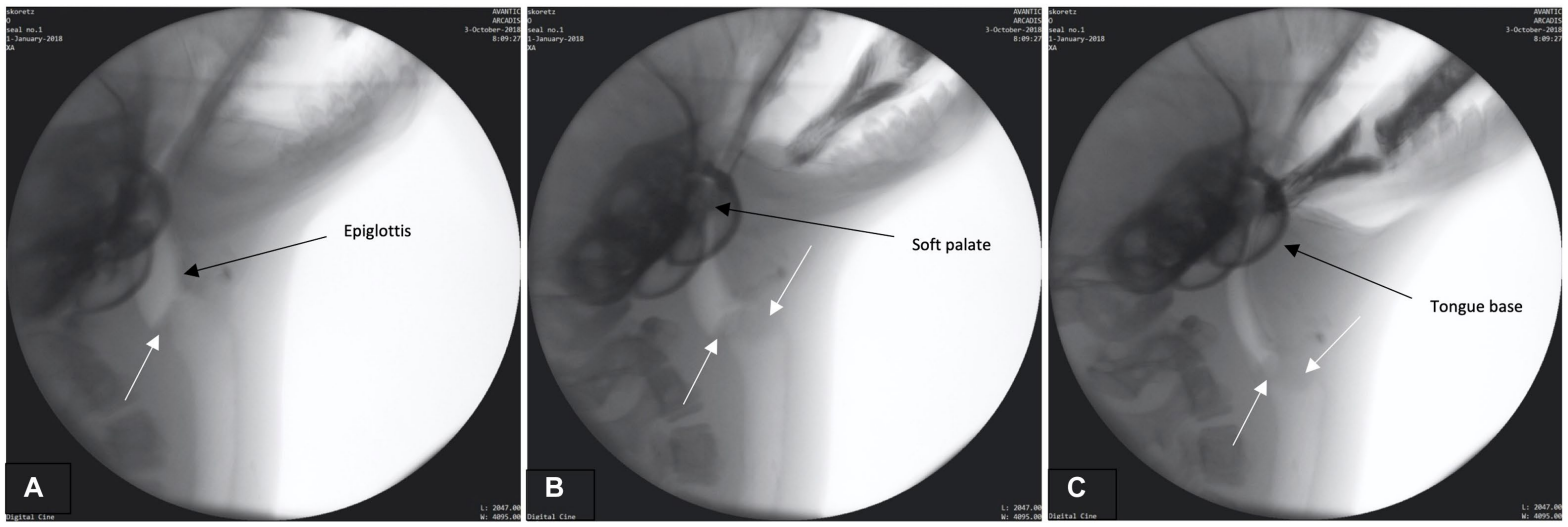
**(A–C)** Fluoroscopic images from the preparatory phase. White arrows indicate modified corniculate cartilage shown moving rostrally **(A)** and approximating glottis **(B,C)**. Other structures (i.e., epiglottis, soft palate, tongue base) also labeled.

The preparatory phase is characterized by two main events: (1) early airway closure and (2) base of tongue (BOT) positioning. Airway closure likely occurred as the boluses were brought toward the seal but prior to the bolus being secured and/or entering into the oral cavity (prehension – please see Supplemental B (Video S1) for slow motion video: Airway closure in seal during preparatory phase). The airway appeared to valve (close) at multiple anatomic levels prior to bolus entry into the oral cavity (Figure 1A). This closure occurred as the: (1) proximal aspect of modified corniculate cartilages appeared to deflect/invert and move rostrally to meet the glottal folds, (2) glottis and corniculates presumably medialize/close as both are bilateral structures (not directly observable in lateral radiographic imaging), and (3) base of tongue (BOT) retracts with depression of the soft palate, closing the caudal aspect of the oral cavity and the narrowing pharyngeal space (Figures 1A–C). Throughout this phase the epiglottis remains upright.

### 3.2 Prehension phase

**Figure 2:**
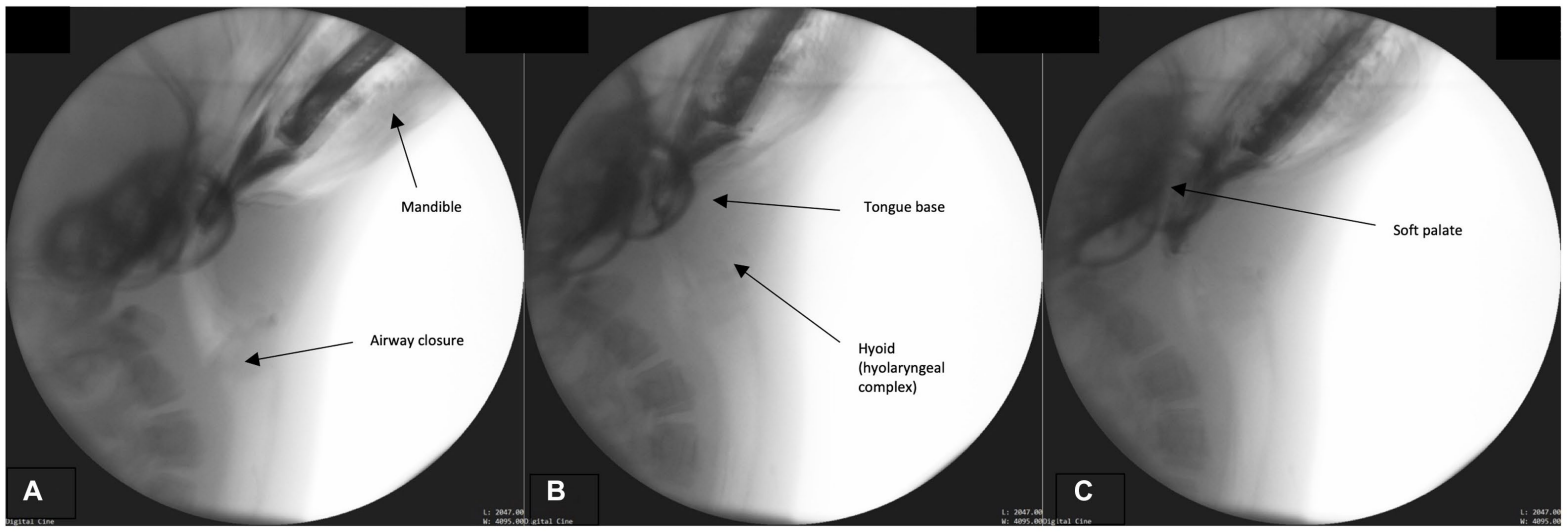
**(A–C)** Fluoroscopic images from the prehension phase. Key events for this phase include continued airway closure (approximation of the modified corniculate cartilages and glottis), mandible elevation, tongue base descent, hyoid (hyolaryngeal complex ascent), and soft palate elevation.

In order to properly position the fish for subsequent swallowing phases, the bolus/prey must be secured and positioned for transport. Please see Supplementary Figure C: Figure S2 for a structural schematic overlaid on a fluoroscopic image from this phase. While the airway remains closed and the head angled toward the prey (Figure 2A), the jaw closes, securing the bolus (Figures 2B,C). No mastication occurs however biting occurs in order to position the bolus head first in the oropharynx and contain it in the oral cavity. The BOT retracts fully into the pharynx and the lateral and posterior pharyngeal wall contracts resulting in maximum circumferential pharyngeal constriction as observed through the contact of the pharyngeal walls on the bolus (Figures 2B,C). Simultaneously, the hyoid bone, along with the larynx (hyolaryngeal complex), begins a rostral ascent under the tongue base, the soft palate begins elevation, and the epiglottis begins to retroflex or invert over the larynx.

### 3.3 Oropharyngeal phase

**Figure 3:**
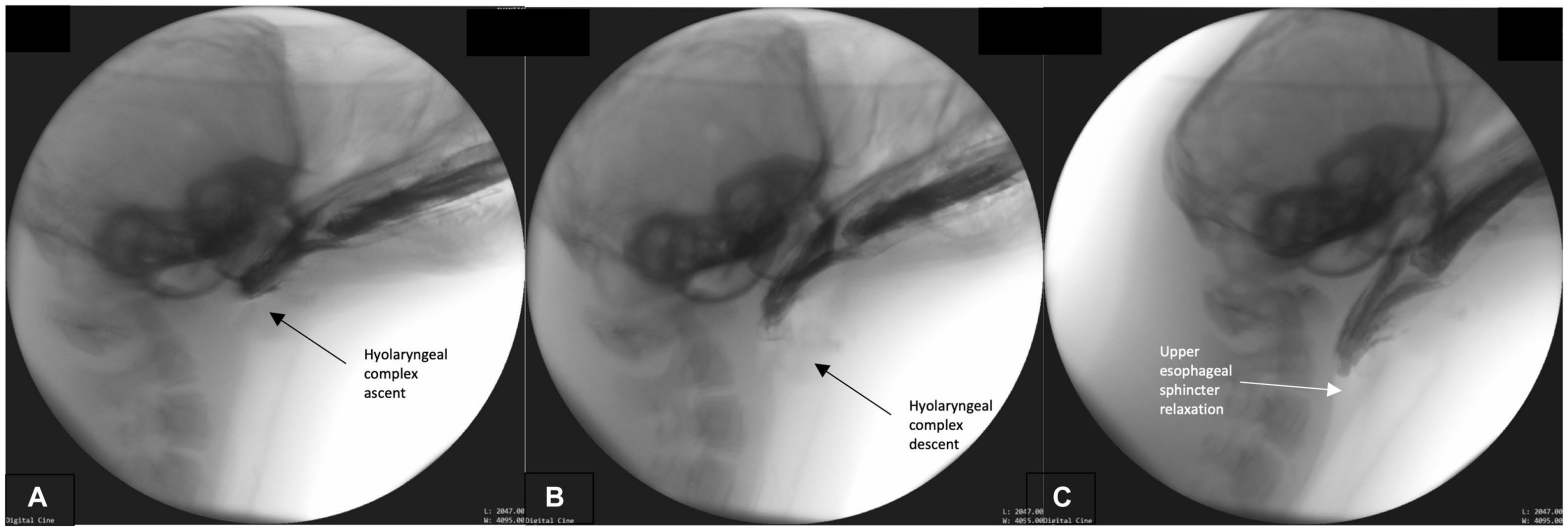
**(A–C)** Fluoroscopic images from the oropharyngeal phase. Rhythmical hyolaryngeal ascent **(A)** and descent **(B)** occur during this phase supporting pharyngeal transport of the bolus. Pharyngeal and tongue base contact on bolus is continual throughout this phase and upper esophageal sphincter relaxation commences **(C)**.

This phase primarily involves bolus transport from the oral cavity, through the pharynx and into the upper esophagus. Bolus transport occurs following BOT retraction, repeated rostral-caudal hyolaryngeal excursion, and pharyngeal wall contraction. To accomplish these maneuvers, the head tends to lower to a neutral rather than extended position. The hyoid and head engage in repeated rhythmical motion which cycles from maximal rostral-caudal hyolaryngeal ascent (Figure 3A) and then returns to rest in a more ventral position (Figure 3B). Simultaneously, pharyngeal constriction and BOT retraction are maintained, as evidenced by the lack of air space between these structures and the bolus (Figure 3C). This likely exerts force and pressure on the bolus propelling it toward the upper esophageal sphincter. As this occurs, the upper esophageal sphincter relaxes in order to accommodate the oncoming bolus (Figure 3C). Airway closure is maintained throughout this phase.

### 3.4 Esophageal phase

**Figure 4:**
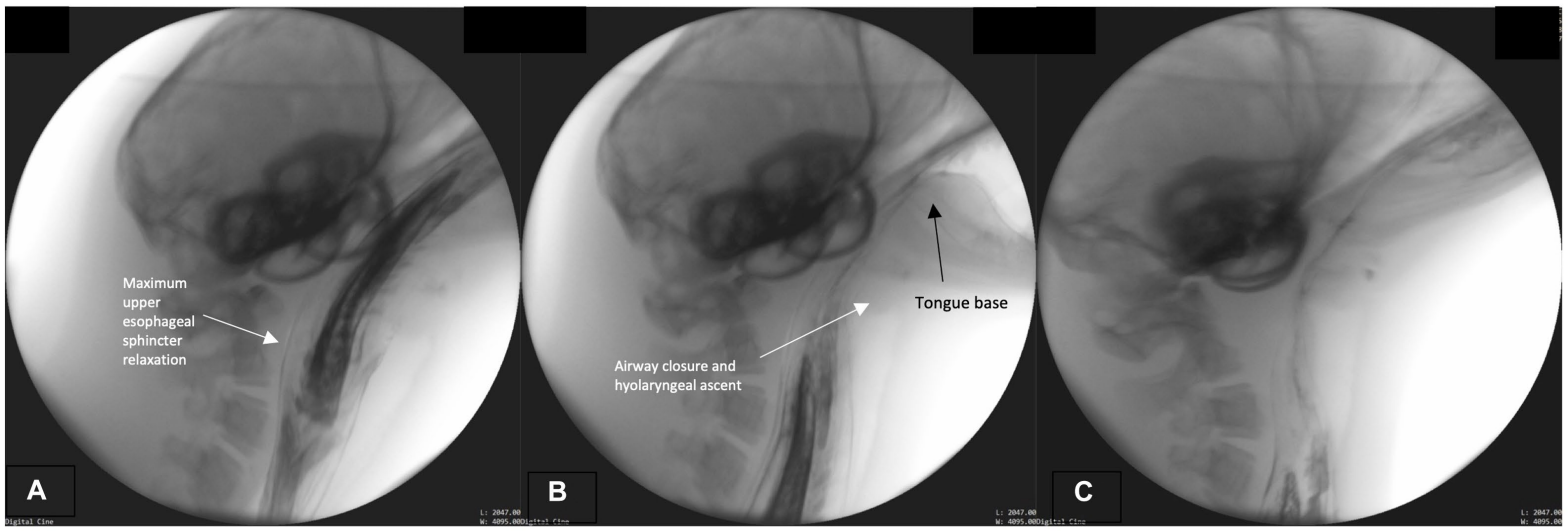
**(A–C)** Fluoroscopic images from the esophageal phase. The upper esophageal sphincter maximally relaxed (distended; **A**). The airway remains closed and tongue base retracted (on bolus tail; **B,C**).

We marked the initiation of this phase when maximum upper esophageal sphincter distention occurs. The bolus passes whole through the distended sphincter into the esophagus, while the hyolaryngeal complex continues its cyclical ascent and descent (Figure 4A). Throughout, the pharynx remains contracted around the bolus until it passes through pharynx and fully enters the esophagus. As the bolus continues to move caudally through the esophagus and the fish tail leaves the sphincter (Figure 4B), the airway continues to remain closed and the epiglottis retroflexed (Figures 4A–C). Upper aerodigestive tract structures return to their rest position after the bolus has entered the distal esophagus. See Supplemental D (Video S2) for a sample harbor seal videofluoroscopic swallowing study.

## 4 Discussion

We have successfully conducted videofluoroscopic swallowing studies on soon-to-be released rehabilitated seal pups, offering a novel glimpse of swallowing physiology. In doing so, we have determined that *in vivo* swallowing imaging is feasible using our protocol, reporting our findings regarding seal swallowing physiology for the first time in their entirety. Furthermore, our preparation of whole herring injected with the barium contrast made the bolus readily observable throughout the seal digestive tract, allowing for deglutition event observations relative to bolus position. Not only was the oral bolus easily visible into the distal esophagus and stomach, the boluses were readily consumed by the seals. Additionally, we are the first to describe four functionally distinct swallow phases, furthering our understanding of airway protection during swallowing in independently feeding seals. In this paper, we have proposed how the paired, modified corniculate cartilages described anatomically in our earlier work (21) may be involved in airway protection during swallowing-as an auxiliary valving mechanism of the seal larynx during swallowing. It remains to be determined if this potential valving adaptation is unique to harbor seals, whether it is crucial in diving and/or if it is a common feature among pinnipeds.

We have taken an innovative perspective, elucidating similarities between harbor seal and human deglutition during swallowing. These include: (1) tongue base retraction and soft palate depression supporting bolus entry strictly to the oral cavity while closing off the pharynx, (2) pharyngeal constriction enabling bolus propulsion to the esophagus, and (3) hyolaryngeal complex elevation supporting airway protection and bolus transition from the oropharynx into the esophagus. While well known in other mammals (36), we are also the first to describe positional changes of the soft palate in harbor seals during swallowing – where the soft palate moves from contact with the tongue base to an elevated position closing off the nasopharynx. With age-appropriate adaptations, this protocol would likely be suitable to gather the first normative swallowing data for neonatal, infant and juvenile seals. This information would enhance our understanding of swallowing physiology in seals, support the development of a cross species swallowing framework, and also provide opportunities to improve diagnostic capabilities and rehabilitation for these animals. Translational research involves bridging basic science to clinical research in order to advance human medicine (37). Although the contemporary focus is shifting from the use of animal models to leveraging biobanking and analysis of human tissue and other samples in “bench” science (38), the use of animal models to inform medical advances in animal (39) and human medicine is longstanding (37). While it is still unknown whether advances made in human medicine could be used in seals, we have emerging information to support a “reversal” in knowledge translation. Not only was this imaging approach successfully used for seals, but once more information is gathered on seal swallowing physiology, intervention approaches used in human medicine may also be translatable.

Although we are the first to describe swallowing in harbor seals using real time, *in vivo* radiographic imaging, others have: described swallowing in other mammals using videofluoroscopy (27–30) and developed predatory aquatic mammal feeding frameworks, with a primary focus on processes related to securing prey (23, 25) and generally applicable mammalian swallowing models (e.g., the Process Model) (40). Hocking and colleagues’ proposed framework included (25): prey capture, prey manipulation and transport, prey processing, water removal, and swallowing. In this work, swallowing is described as the passage of food through the digestive tract, and specific to seals, transported by serial “gulping” (p. 5). Kienle and colleagues proposed a modified version of this framework (23) to provide more flexible application across aquatic mammals when compared to other tetrapods. The phases were as follows: ingestion, intraoral transport, processing, water removal, and swallowing. In this adapted framework, “ingestion encompasses all behaviors used to capture, subdue, kill and process prey before it enters the oral cavity” (p. 1). This previously published work also suggests that these phases may be modulated according to feeding event. Similarly, human swallowing also modulates, despite being described as partially reflexive (41, 42). Based on our current observations, we propose that swallowing events are initiated much earlier within these frameworks than previously described, and therefore, feeding and swallowing processes should be described as overlapping synergistic processes occurring simultaneously. Specifically, we observed airway “valving” (through the approximation of the modified corniculate cartilages and glottis; tongue base and soft palate contact) well before the prey was secured. This adaptation is crucial to the protection of the airway from aspiration, and may contribute to differential pressure generation to facilitate feeding processes (e.g., suction) (26) and swallowing processes (e.g., bolus transport). Specific to bolus transport, we recognize that prehension may vary depending on feeding circumstances, as a result, we propose a broad prehension definition to include specific feeding and/or positioning methods (e.g., grasping, crushing and/or suction) even though some actions were not directly observed (i.e., crushing) or measured (i.e., suction) during our study. The Process Model was designed to explain a variety of mammalian feeding behaviors, specifically for those who consume a variety of bolus texture types (i.e., liquid, semisolid, and solid), and assuming that solids need to be altered in order to be swallowed (40). Although harbor seals primarily consume their solid boluses whole, our observations align with the two transport types proposed in this model (40). Specifically, Stage I transport is described as when the bolus is “moved from the incisal area to the postcanine region (p. 417)” – which was observed during our prehension phase. Stage II transport occurs when the bolus is positioned and the swallow is initiated – aligning with our oropharyngeal phase.

In all terrestrial vertebrates, the lower respiratory tract develops early in gestation as an outgrowth of the primitive ‘gut tube’. As a result, mammalian breathing and swallowing share a common pathway – the pharynx – which predisposes them to adverse outcomes (e.g., airway invasion/aspiration) should a food/liquid bolus be misdirected away from the esophagus and into the airways (43). The larynx and other AT structures engage in “valve-like” movements to facilitate this separation as the bolus leaves the oral cavity (44). In this study, we have observed the potential function of paired modified corniculate cartilages at the laryngeal inlet, which when oral intake is anticipated, approximate the glottis or vocal folds. This airway “valving” or covering of the laryngeal inlet was observed early in the “preparatory phase” during which the oral boluses were transported (or proffered) toward the seal and imaging equipment. Although our study was conducted using dry conditions without submersion of either the seal or oral bolus, we hypothesize that this adaptation, along with valving by the soft palate to tongue base, are early and pre-emptive mechanisms to protect the airway during submerged feeding, reaffirming our earlier anatomical descriptions (21). During the “prehension phase” of underwater feeding, if the seal opens the oral cavity without these protectionary valving mechanisms, the airway would be exposed to water influx. This same valving mechanism may also be engaged during deep dives as an adaptation to prevent aspiration of water into the airway under pressure. In humans, these movements include similar protective mechanisms: (1) tongue base to soft palate contact and then soft palate elevation/tongue base depression, protecting the airway and nasal passages, respectively, from oncoming bolus, and (2) anterior/superior hyolaryngeal displacement for airway closure including vocal fold and aryepiglottic folds medialization and epiglottic retroflection (44).

Although outside of the scope of our feasibility study, some investigators have described morphological adaptations for feeding (45, 46) while others have explored feeding kinematics including pressure measurements (26, 47, 48). Harbor seals have been reported to engage in both pierce (46) and suction feeding (26). Harbor seals also share cranial morphologic characteristics with other pinnipeds including harp seals, ribbon seals, and Ross seals (49), particularly “short (rostro-caudally) and narrow (medio-laterally) caudal portions of the skull, wide (medio-laterally) rostra, thickened (dorso-ventrally) palates” p. 402 (49). Mandibular girth (49) and specialized dentition (45) enable prey piercing or biting (49) however, harbor seals are also able to engage in suction feeding without specialized skull morphology or ability to generate relatively high subambient pressures as compared to other pinnipeds (50). In humans, pressure differentials occur during the swallow where the bolus moves from areas of higher pressure (e.g., in the oral cavity, pharynx) to areas of relatively lower (or subambient) pressure (e.g., the esophagus) (51). These pressure differentials are generated even out of the context of volitional sucking, in part due to increased lingual pressure placed on bolus and “valving” of the base of tongue to soft palate during bolus preparation (52). When the bolus is compressed and ready for transport into the pharynx, the base of tongue lowers and soft palate raises, creating an area of lower pressure relative to that of the oral cavity, with pressure on the bolus increased again with pharyngeal contraction (51). Harbor seals engage in suction feeding despite the previously reported lack of unique cranial adaptations to do so (50). We hypothesize that suction may also be supported by “valving” through the creation of an area of relatively lower pressure, or subambient pressure specifically during: (1) early “valving” of the modified corniculate cartilage and glottis, and (2) soft palate depression and tongue base retraction closing off the pharynx. Once the prey is secured and oral transport initiated, the tongue base to palate “valve” is opened and an area of subambient pressure is generated – potentially creating, or supporting, suction.

Current interventions for feeding and swallowing in seals and humans have had different foci. In seals, feeding enrichment intervention supports foraging development (e.g., underwater feeding boxes) and swimming skills (e.g., floating kelp) (53). We are aware of only one controlled study (54) where clinical outcomes were compared between seals randomized to enrichment versus standard rehabilitation care. In that study, progression to independent feeding was not expedited in those receiving enrichment however, improved foraging was observed (31). Human infant feeding and swallowing rehabilitation include enrichment but also bridging therapies for those transitioning from tube feeding to oral intake (55). In a metanalyses of randomized controlled trials (56), preterm human infants receiving oral motor intervention had shorter transition time to full oral intake, shorter hospitalizations, more weight gain, and improved feeding efficiency. The incorporation of non-nutritive sucking along with oral motor interventions has also improved outcomes (32, 57). While facilitating spontaneous sucking is not a typical intervention focus in seals, some engage in spontaneous sucking behavior using their flipper or tub wall (58). Similarly some rooting behaviors were noted in the seals with stimulation to the external buccal area, as observed in human neonates (59). While investigations focused on developing oral feeding devices to support seal feeding and swallowing rehabilitation are few (11, 31) and their effectiveness is emerging, given the promising results in human preterm infants, translating these advances should be explored for seal rehabilitation.

By virtue of its feasibility design, this study has inherent limitations. We conducted the protocol with only two seals, and while we are confident that the observations are generalizable to other seals of similar age, we were not able to confirm its application to younger seals and/or those who are not feeding independently. Additionally, this study did not explore feeding strategies and/or kinematics of aerodigestive tract structures. Because of the novelty of this endeavor, we did not attach a known scalar to the seals during imaging. Doing so would have afforded us the opportunity to conduct structural movement analyses, documenting individual variations during swallowing. This information would not only contribute normative group data but also provide information on swallowing modulation in response to changes in environmental conditions (e.g., bolus size). Additionally, we did not conduct durational measures due to our small sample size as a larger data set would provide a more in-depth characterization of swallowing events. Our dry environment and the few analyzable swallows available also limited our study. As this study was to determine the feasibility of performing a videofluoroscopic swallowing study in seals, we elected not to include a submerged condition thereby limiting what we could explore about: (1) natural head positioning during prehension, (2) feeding or “ingestion” strategies, (3) the influence of suction on the swallow, and (4) how water is evacuated from the oropharynx prior to movement of the bolus into the esophagus. We are pursuing measures to explore an aquatic environment for videofluoroscopy as well as improve our movement tracking to increase the number of analyzable events. Finally, this study was completed on seal pups who were successfully rehabilitated and ready for release to the wild. It remains to be determined whether or not our physiological findings would be generalizable to free ranging seals. With the successful completion of this study and exploration of seal imaging techniques, we are well positioned to continue our mechanistic work.

Understanding how seals swallow while integrating airway protection provides further insight into mechanisms, adaptations, and biological modulation while determining translatable applications between terrestrial mammal and seal species. Future work should focus on detailed laryngeal imaging, kinematic movements of seal upper aerodigestive tract structures, comparisons of these structural movements during oral intake on land and underwater, and whether swallowing changes in seals during early development or at the time of weaning. This information will contribute to the development of dynamic multi-system assessment approaches and determine feasible rehabilitation translation between species, specifically compensatory processes to support an individual’s resumption of ‘natural’ ways of eating. With thousands of abandoned seals admitted annually around the world for rehabilitation, future work will inform biomechanical interventions and determine optimal timing for their use. Regardless of species, protecting the lower airway from food/liquid will reduce adverse events and system burden while improving life quality – important during this time of limited health and human resources.

## Data availability statement

The raw data supporting the conclusions of this article will be made available by the authors, without undue reservation.

## Ethics statement

The animal study was approved by the University of British Columbia Animal Care Committee. The study was conducted in accordance with the local legislation and institutional requirements.

## Author contributions

SS: Conceptualization, Formal analysis, Funding acquisition, Investigation, Methodology, Project administration, Resources, Visualization, Writing – original draft, Writing – review & editing. AA: Formal analysis, Methodology, Validation, Writing – original draft, Writing – review & editing. AV: Formal analysis, Methodology, Validation, Writing – review & editing. SR: Methodology, Validation, Writing – review & editing. MH: Investigation, Methodology, Project administration, Supervision, Writing – review & editing. HS: Formal analysis, Validation, Writing – review & editing. CD: Conceptualization, Formal analysis, Methodology, Validation, Writing – review & editing.
