# Supplementary figures and images for: Videofluoroscopy of the aerodigestive tract in Phoca vitulina: reshaping perspectives on translational medicine
Source: Front Vet Sci. 2024 Jul 18;11:1412173. doi: 10.3389/fvets.2024.1412173 (PMC11291318; doi:10.3389/fvets.2024.1412173)

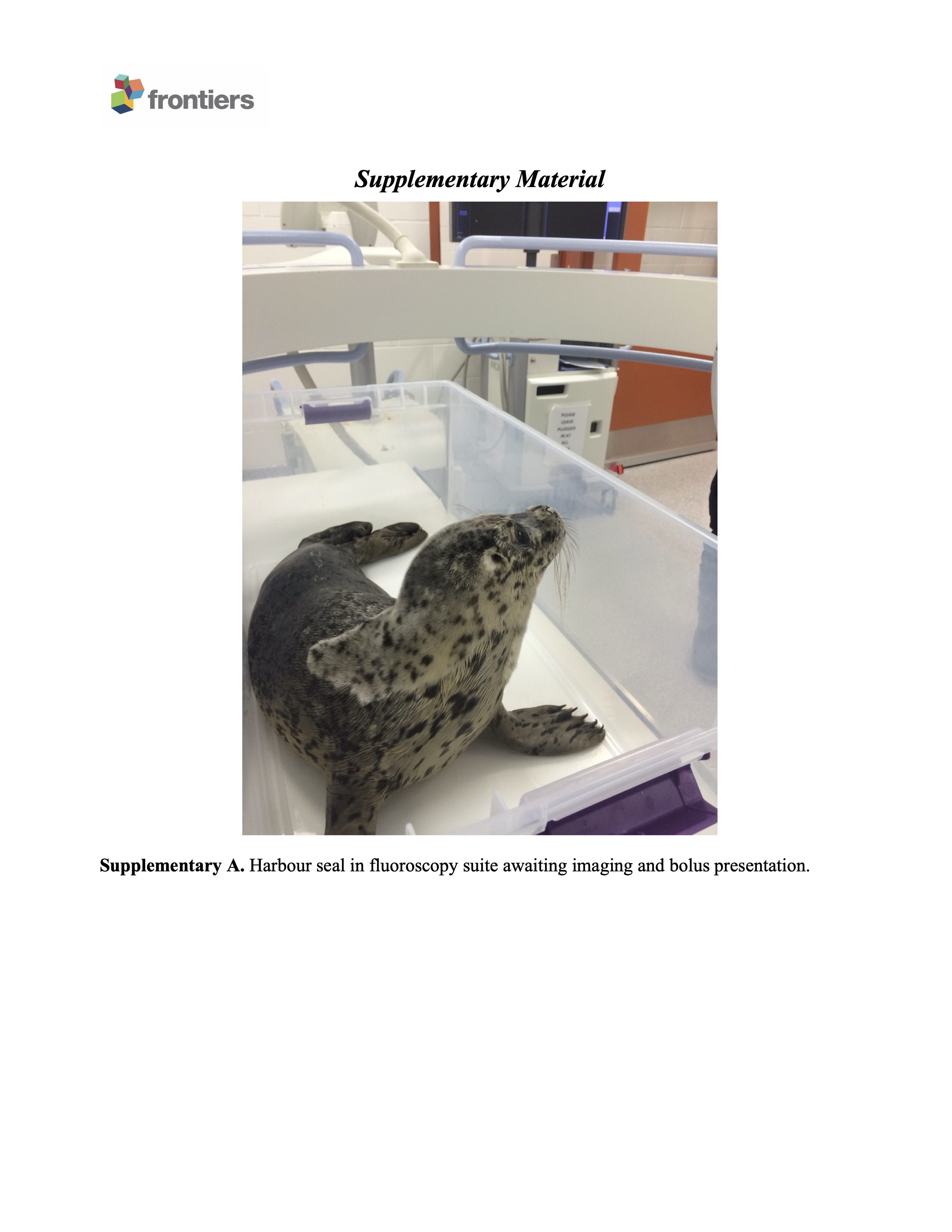

Supplement: Supplementary file 3 [file Image_1.JPEG]

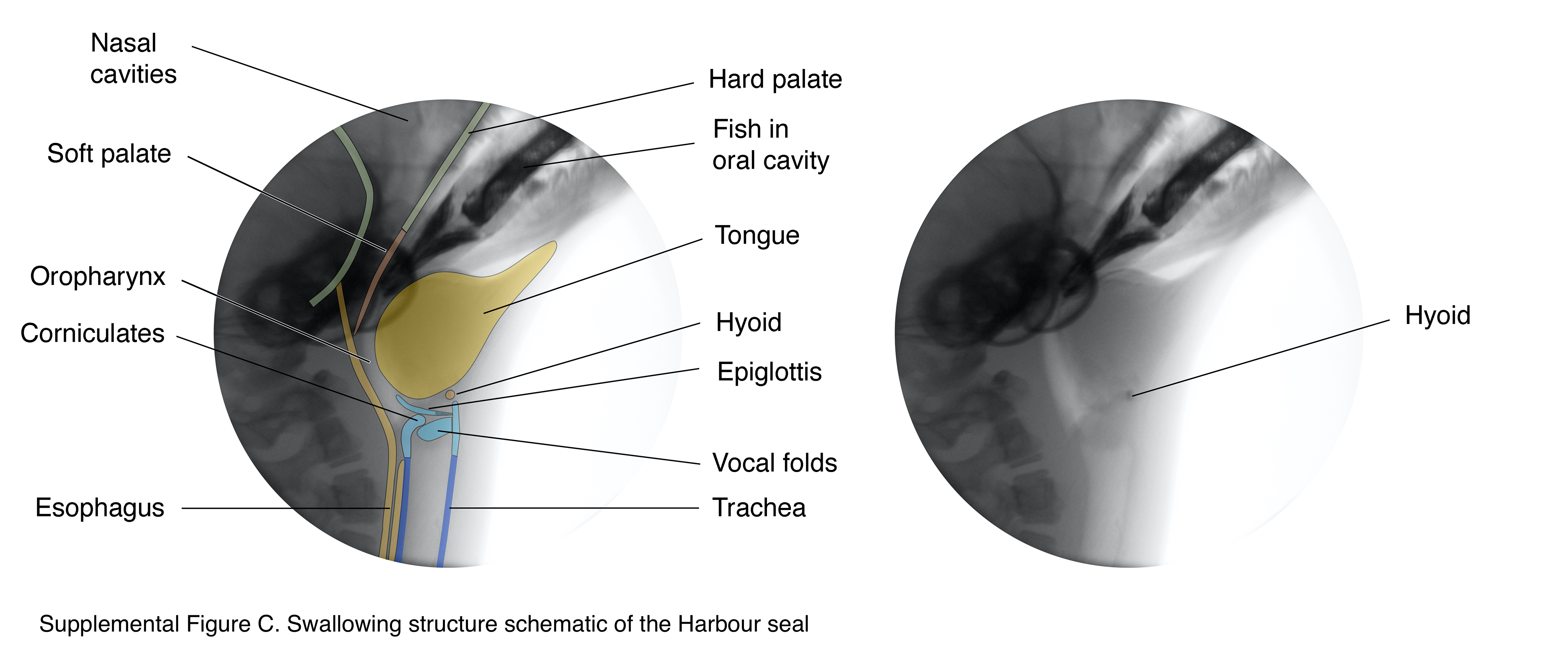

Supplement: Supplementary file 4 [file Image_2.JPEG]
